# Activation of Akt is increased in the dysplasia-carcinoma sequence in Barrett's oesophagus and contributes to increased proliferation and inhibition of apoptosis: a histopathological and functional study

**DOI:** 10.1186/1471-2407-7-97

**Published:** 2007-06-08

**Authors:** Ian LP Beales, Olorunseun Ogunwobi, Ewen Cameron, Khalid El-Amin, Gabriel Mutungi, Mark Wilkinson

**Affiliations:** 1 Gastroenterology Unit, Norfolk and Norwich University Hospital, Norwich, NR4 7UZ, UK; 2 Department of Cell Biology and Physiology, School of Medicine, Health Policy and Practice, University of East Anglia, Norwich, NR4 7TJ, UK; 3 Department of Histopathology, Norfolk and Norwich University Hospital, Norwich, NR4 7UZ, UK

## Abstract

**Background:**

The incidence of oesophageal adenocarcinoma is increasing rapidly in the developed world. The serine-threonine protein kinase and proto-oncogene Akt has been reported to regulate proliferation and apoptosis in several tissues but there are no data on the involvement of Akt in oesophageal carcinogenesis. Therefore we have examined the activation of Akt in Barrett's oesophagus and oesophageal adenocarcinoma and the functional effects of Akt activation *in vitro*.

**Methods:**

Expression of total and active (phosphorylated) Akt were determined in endoscopic biopsies and surgical resection specimens using immunohistochemistry. The functional effects of Akt were examined using Barrett's adenocarcinoma cells in culture.

**Results:**

In normal squamous oesophagus, erosive oesophagitis and non-dysplastic Barrett's oesophagus, phospho-Akt was limited to the basal 1/3 of the mucosa. Image analysis confirmed that Akt activation was significantly increased in non-dysplastic Barrett's oesophagus compared to squamous epithelium and further significantly increased in high-grade dysplasia and adenocarcinoma. In all cases of high grade dysplasia and adenocarcinoma Akt was activated in the luminal 1/3 of the epithelium. Transient acid exposure and the obesity hormone leptin activated Akt, stimulated proliferation and inhibited apoptosis: the combination of acid and leptin was synergistic. Inhibition of Akt phosphorylation with LY294002 increased apoptosis and blocked the effects of acid and leptin both alone and in combination. Activation of Akt was associated with downstream phosphorylation and deactivation of the pro-apoptotic protein Bad and phosphorylation of the Forkhead family transcription factor FOXO1.

**Conclusion:**

Akt is abnormally activated in Barrett's oesophagus, high grade dysplasia and adenocarcinoma. Akt activation promotes proliferation and inhibits apoptosis in Barrett's adenocarcinoma cells and both transient acid exposure and leptin stimulate Akt phosphorylation. Downstream targets of Akt include Bad and Forkhead transcription factors. Activation of Akt in obesity and by reflux of gastric acid may be important in the pathogenesis of Barrett's adenocarcinoma

## Background

The incidence of oesophageal adenocarcinoma (OAC) is continuing to increase in the developed world. Over the last 30 years the incidence in the USA has increased six fold [1]. Most cases of OAC are believed to develop from the precursor lesion, metaplastic glandular oesophageal epithelium (Barrett's oesophagus, (BO)), evolving through a sequence from low grade, to high grade dysplasia (HGD) and eventually to carcinoma, yet the factors which drive the progression are incompletely understood [2,3]. In view of the poor prognosis of OAC and the risk of malignant transformation, regular endoscopic biopsy screening of confirmed BO has been advocated with ablative therapies or oesophagectomy advised if high grade dysplasia is detected [4]. This approach remains controversial because of doubts about the cost-effectiveness of screening biopsies in this population, where the rate of progression to malignancy is still relatively low (estimates suggest 1 in 50–200 per year) whilst the diagnosis of HGD from limited biopsies can be extremely difficult [5-8].

A greater understanding of the biology of BO is needed to provide us with more targets for preventative and therapeutic interventions as well as markers of progression in BO, to enable more focused screening. Although several genetic and cellular changes have been described, none of these as yet have proven utility [4,9]. Increased proliferation and decreased apoptosis are hallmarks of metaplastic Barrett's oesophagus: these changes are believed to be important in malignant progression by increasing the vulnerability to, and perpetuation of mutations [2,3]. Again the factors driving these changes and the cellular pathways involved are not completely defined.

The protein kinase Akt (also know as protein kinase B) is a relatively recently described serine-threonine kinase that has been shown to be important in mediating cell proliferation and cell survival signals in several tissues and cancers [10,11]. There are only limited data on Akt activation in BO: studies have suggested that gastrin-mediated proliferation in oesophageal adenocarcinoma and Barrett's oesophagus is Akt-dependant [12,13]. Pharmacological inhibition of phosphoinositol-3'kinase (PI3-kinase), which is a potential upstream activator of Akt, has been shown to reduce proliferation and induce apoptosis in cultured oesophageal cancer cell lines, without specifically examining the involvement of Akt [14]. There are no data concerning Akt activation in the Barrett's metaplasia-carcinoma sequence.

In this study we have examined the activation of Akt using immunohistochemistry in biopsies from the spectrum of histological progression from normal to Barrett's oesophagus to adenocarcinoma. We have then examined the functional effects of Akt activation *in vitro *using the OE33 and OE19 Barrett's-derived adenocarcinoma cell lines. These have previously been shown to be suitable functional experimental cellular models for Barrett's epithelium [13,15]. We have used short-term acid exposure and leptin as experimental upstream activators to test involvement of the Akt pathway. The two main risk factors for OAC are oesophageal acid reflux and obesity [16-19]. Previous studies have shown that intermittent acid exposure stimulated proliferation and inhibited apoptosis in BO and OAC cell lines, although the involvement of Akt has not been examined [20-23]. Serum levels of the peptide hormone leptin are increased in obesity in proportion to body fact mass [24], recent data suggest that leptin receptors are expressed by BO and OAC [25,26] and studies have also shown that leptin can stimulate proliferation of OAC cells in culture [26,27]. Leptin stimulated proliferation and ameliorated serum-starvation and celecoxib-induced apoptosis in HT-29 colon cancer cells via Akt activation [28]. Akt has been reported to phosphorylate and subsequently regulate the function of several downstream effectors: potential downstream targets of Akt include the pro-apoptotic protein Bad, which is inactivated by Akt-mediated phosphorylation [29,30] and the Forkhead (FHKR) family transcription factors [31,32]. Akt-mediated phosphorylation of these transcription factors promotes their extrusion from the nucleus and this resulting alteration in gene transcription may be important in the regulation of cell proliferation. Therefore to test the integrity of the PI3-kinase/Akt pathway we have examined the activation of Bad and the Forkhead transcription factor FOXO1.

## Methods

### Patients

Histopathology specimens were examined from patients under the care of the Gastroenterology unit at the Norfolk and Norwich University Hospital. All patients gave informed consent for the use of their histology blocks in the research study. The local research ethics and research and development committees approved the study. (projects 2003/084 and 2003/085). Tissue samples from both endoscopic and oesophagectomy specimens were examined. The diagnosis of high grade dysplasia (without cancer) was confirmed by at least 2 independent gastrointestinal pathologists. The presence of endoscopic oesophagitis was scored according to the Los Angeles System [33] and for the sake of clarity all endoscopic biopsies used in the study were taken from segments of columnar-lined oesophagus at least 2 cm in length. A total of 40 patients were studied: 4 normal, 8 with erosive oesophagitis (Los Angeles C or D grade), 13 with non-dysplastic Barrett's oesophagus, 4 with high grade dysplasia, 11 with adenocarcinoma.

### Immunohistochemistry

5 μM Sections of formalin-fixed, paraffin-embedded samples were dewaxed in xylene and rehydrated. Antigen retrieval was performed by 2 minutes microwave heat treatment in 10 mM sodium citrate buffer pH 6.0. Endogenous peroxidase was blocked in 3% hydrogen peroxide in 70% methanol for 20 minutes. Non-specific binding was blocked with 3% goat serum for 30 minutes. Primary antibodies (rabbit polyclonal anti-Akt and anti-phospho-(serine 473)-Akt, both from Cell Signalling Technology, Hertfordshire, UK), were used at 1/50 and sections incubated for 60 minutes at room temperature. Detection was with a biotin-conjugated goat anti-rabbit secondary antibody (Santa Cruz Biotechnology, Santa Cruz, CA) used at 1/400 and the Vector Elite ABC detection kit (Vector Laboratories, Burlingame, CA, USA) with 3,3'-diaminobenzidine tetrahydrochloride (Sigma) as the chromogen. Sections were counterstained with Mayer's haematoxylin.

### Quantitation of Akt phosphorylation

Initial qualitative analysis of the slides by two independent observers (MW and KE) without knowledge of the clinical details revealed obvious differences in the patterns of phospho-Akt staining. Subsequently the density of staining was quantified on digitised grey-scale images of the slides using the free NIH Image for PC software using the standard protocol (Scion Image^®^). The relative staining densities in the basal and luminal thirds of the epithelium of each subject were determined by taking the mean pixel grey-scale density value from 5 separate equal area fields in the relevant areas.

### Cell culture, proliferation and apoptosis

The Barrett's adenocarcinoma derived OE33 and OE19 cell lines were obtained from the European Collection of Animal Cell Cultures (Wiltshire, UK) and subcultured as previously described [15]. Proliferation studies were performed using the MTT (3- [4, 5-dimethylthiazol-2-y-l]-2, 5-diphenyltetrazolium bromide, Sigma) and BrdU incorporation (Roche, Mannheim, Germany) assays and apoptosis was quantified using an both an ELISA for intracellular nucleosomes (Roche) and an ELISA for caspase-3 activity in cell lysates (R and D Systems, Abingdon, UK) as described previously [23,26,35,36]. To examine the effects of the PI3-kinase/Akt pathway cells were incubated with the PI3-kinase inhibitor LY294002 (Alexis Biochemicals, Nottingham, UK) for 60 minutes before stimulation [26]. To test the effects of stimulation of the Akt pathway cells were serum-starved for 24 hours before treatment with recombinant human leptin (Bachem, St Helens, UK) and/or transient acid exposure and cell numbers or apoptosis assessed after 24 hours. For acid-pulse experiments, serum starved cells were exposed to serum-free culture medium at pH 4.0 for 3 minutes at 37°C, before replacing the media with standard serum-free medium supplemented where appropriate with leptin [23]. Control cells were identically treated with standard serum-free medium for the 3 minute period.

### Immunocytochemistry

OE33 cells were air-dried and fixed in 4% formaldehyde for 10 minutes at room temperature. After washing 3 times in phosphate buffered saline (PBS) cells were permeabilized with 0.2% triton X-100 for 5 minutes at room temperature, followed by 3 further PBS washes. Non-specific antibody binding sites were then blocked with 1% rabbit serum for 45 minutes. Cells were incubated in 1:200 goat polyclonal anti-leptin receptor antibody (M-18 Santa Cruz) in 1% BSA in PBS overnight at 4°C. Primary antibody binding was recognised by incubating with 1:400 donkey anti-goat FITC conjugated secondary antibody (Santa Cruz) for 30 minutes at room temperature, followed by visualisation with a Zeiss Axioplan 4 CCD Upright fluorescent microscope equipped with AxioVision digital imaging software. Images were acquired at × 63 magnification.

### RNA extraction and reverse transcriptase polymerase chain reaction (RT-PCR)

Total RNA was isolated from OE33 cells using a one-step RT-PCR kit (Qiagen). Primers for the long (Ob-Rb), short (Ob-Ra) and common sequence leptin receptors were obtained from Invitrogen, using previously published primer sequences [26]. Reverse transcription comprised incubation at 50°C for 30 minutes and at 95°C for 15 minutes. PCR was performed for 40 cycles comprising a 60-second denaturation step at 94°C, a 60-second annealing step at 55°C, a 90-second extension step at 72°C followed by 10 minutes at 72°C. Electrophoresis on a 1.8% agarose gel stained with ethidium bromide was used to separate PCR products.

### Protein phosphorylation

OE33 cells were grown in 96 well plates as previously described, stimulated as for the apoptosis and proliferation experiments. Cells were then formalin fixed and phosphorylated and total Akt, Bad and FKHR (FOXO1) were assessed by specific cell based ELISAs (Active Motif, Belgium) as described [35,36].

### Statistical analysis

Proliferation and apoptosis experiments were performed in duplicate wells and protein phosphorylation in quadruplicate. The mean of all wells from a single experiment was regarded as N = 1 and each experiment was repeated 3–6 times. Results were compared to untreated control wells on the same tissue culture plate. Analysis of variance was used to analyse the leptin dose-response curve. Differences in the density of immunohistochemical staining and the responses to different stimulants and agents were compared using Student's t-test, with Bonferroni's correction for multiple comparisons, *P *< 0.05 was regarded as significant.

## Results

### Oesophageal phosphorylated Akt expression

**Figure 1:**
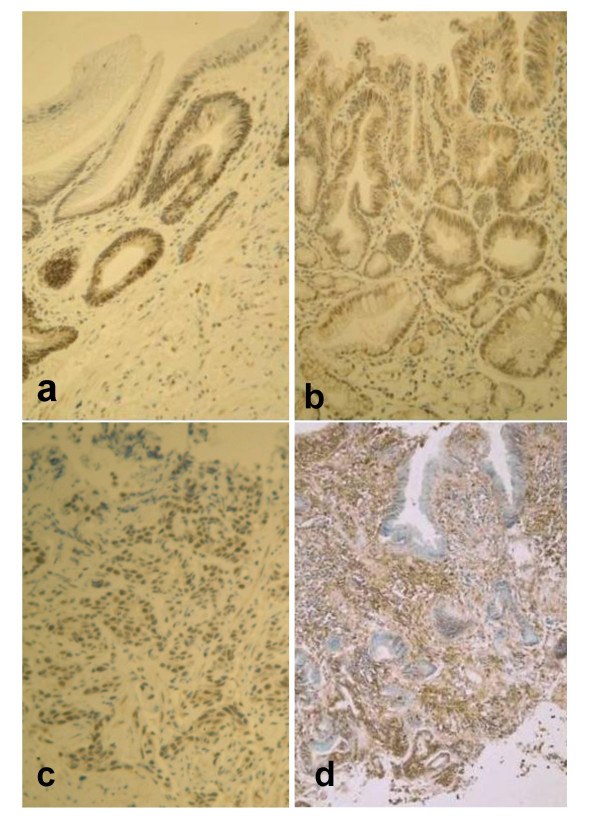
**Immunohistochemistry of activated (phospho-) and total Akt in Barrett's oesophagus. a: **Phospho-Akt localisation in non-dysplastic Barrett's oesophagus showing staining in gland crypts but not surface epithelium (original magnification × 20). **b: **Phospho-Akt localisation in high-grade dysplasia in Barrett's oesophagus with showing staining throughout the epithelium reaching the luminal surface (original magnification × 20). **c: **Phospho-Akt expression in oesophageal adenocarcinoma showing staining throughout the epithelium reaching the luminal surface (original magnification × 20). **d: **Total Akt expression in non-dysplastic Barrett's oesophagus showing considerable staining in epithelial cells in all layers (original magnification × 10).

**Figure 2:**
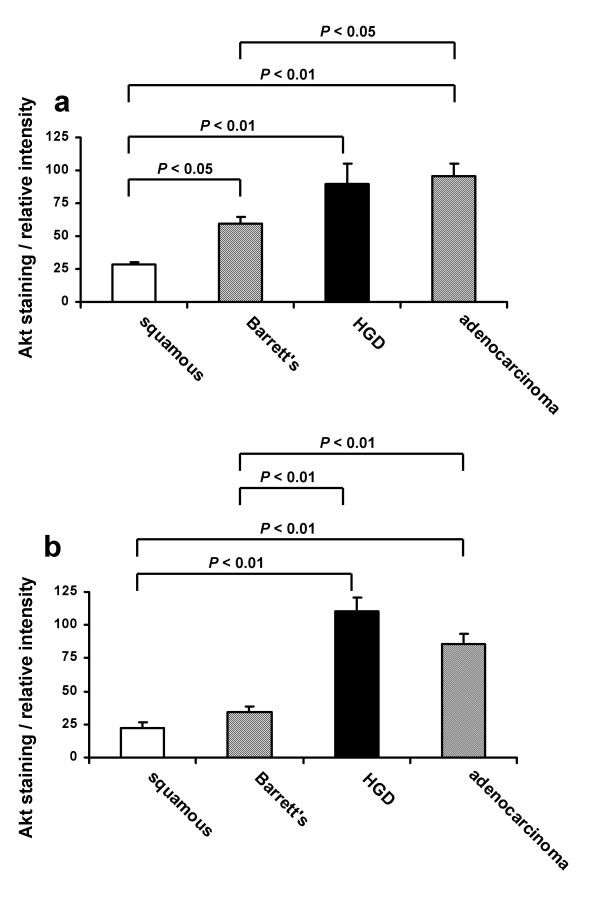
**Intensity of phosphorylated-Akt expression in Barrett's oesophagus**. Relative intensity of phospho-Akt staining was assessed on digital images from squamous epithelium (squamous), non-dysplastic Barrett's oesophagus (Barrett's), Barrett's with high-grade dysplasia (HGD) and adenocarcinoma using Scion Image^® ^Software. **2a **shows the relative intensity of staining in the basal third of the epithelium and **2b **shows the relative intensity of staining in the luminal third of the epithelium. Results expressed as mean ± SEM.

Phosphorylated Akt was detectable by immunohistochemistry in all 40 specimens examined. Initial examination of the slides revealed an obvious qualitative difference in the distribution of phospho-Akt across the epithelium between the different disease types and this was confirmed by image analysis. In normal squamous oesophageal epithelium and erosive oeosphagitis phospho-Akt was present only in the basal (proliferative) layer. In 12/13 cases of non-dysplastic BO phospho-Akt expression was also limited to the crypts in the basal epithelial zone. In contrast in both high grade dysplasia and adenocarcinoma phospho-Akt staining was obvious and prominent in the more superficial epithelial zones reaching the lumen in all cases. Phospho-Akt was located in both the cytoplasmic and nuclear compartments. Dense total Akt expression was seen throughout all epithelial zones in all cases. Figure 1 shows representative immunohistochemical staining patterns. Quantitation using image analysis software showed that the basal third of the epithelium (the crypts) stained significantly more intensely for phospho-Akt in non-dysplastic Barrett's oesophagus compared to squamous epithelium, and both HGD and adenocarcinoma stained significantly more intensely than non-dysplastic Barrett's (fig 2a). Akt activation was comparable in the squamous epithelium of both non-inflamed and oesophagitis cases and all these were included within the squamous group for further comparisons. In the luminal third of the epithelium negligible phospho-Akt staining was seen in both squamous and non-dysplastic Barrett's epithelia but phospho-Akt staining was significantly greater than both of these in both HGD and adenocarcinoma (fig 2b). Staining was if anything less intense in the adenocarcinomas but this was not significantly reduced compared to HGD. Quantitation of control staining with anti-total Akt antibody did not show any significant difference in expression of the total inactive non-phosphorylated protein between the different conditions (data not shown).

### Functional leptin receptor expression

**Figure 3:**
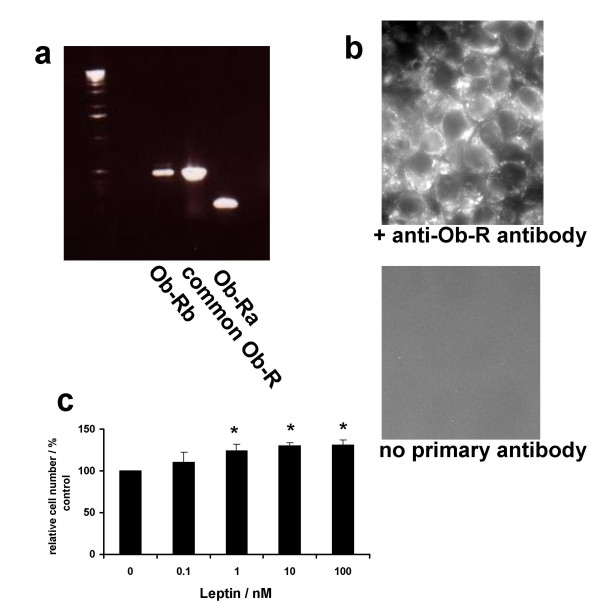
**Functional leptin receptor expression by OE33 cells**. Expression of the long (Ob-Rb), short (Ob-Ra) and common sequence leptin receptors was detected by RT-PCR using specific primers (**3a**). Surface expression of Ob-R was detected using immunocytochemistry with an anti-Ob-R antibody, primary antibody binding was recognised using a donkey-FITC conjugated ant-rabbit antibody and fluorescence microscopy (**3b top panel**). No staining was detected in the absence of anti-Ob-R antibody (**3b bottom panel**) (× 63 magnification). Leptin increased cell numbers in OE33 cells (**3c): **serum-starved OE33 cells were treated with increasing concentrations of leptin and relative cell numbers assessed after 24 hours using the MTT colourmetric assay, results expressed as mean ± SEM, N = 3, *P < 0.05 vs basal.

**Figure 4:**
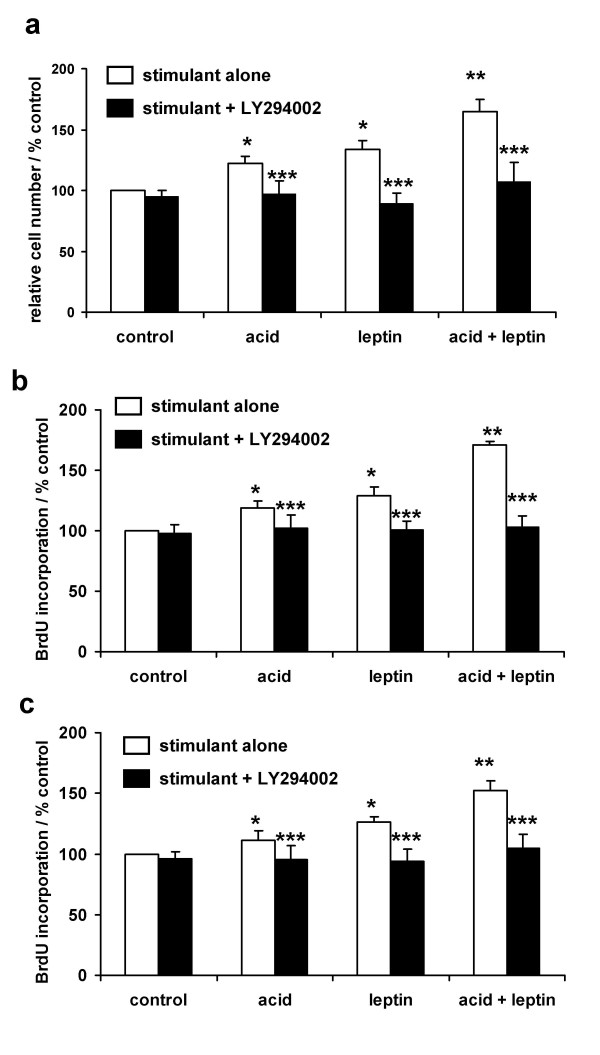
**Akt activation is essential for acid and leptin stimulated proliferation of oesophageal cancer cells**. Serum-starved OE33 cells were treated with a pH 4.0 acid or control pulse for three minutes and/or leptin (10 nM) for 24 hours and then relative cell numbers assessed by MTT assay (**4a**) or DNA synthesis by BrdU incorporation (**4b**). DNA synthesis in similarly treated OE19 cells was assessed by BrdU incorporation (**4c). **Where indicated cells were pretreated with the PI3-kinase inhibitor LY294002 (10 μM) for 60 minutes before any stimulation. Results are expressed as the percentage of value compared to untreated control cells on the same plate. * *P *< 0.05 vs untreated control, ** *P *< 0.05 vs acid or leptin alone, *** *P *< 0.05 vs stimulus in the absence of LY294004, N = 3–6, mean ± SEM.

Leptin receptor expression was detected by at both mRNA and protein level in OE33 cells; mRNA sequences for long, short and common isoforms were detected (fig 3a) and immunocytochemistry showed localisation of leptin receptor to the cell surface (fig 3b). Exogenous leptin significantly increased proliferation in both OE33 and OE19 oesophageal adenocarcinoma cell lines (fig 3c, fig 4a, fig 4b & fig 4c). Maximal effects of leptin were seen with 10 nM and this concentration was used in the mechanistic studies.

### Functional effects of Akt

To assess the functional effects of phospho-Akt in BO and OAC we examined the effects of stimulation or inhibition of Akt activity in OE33 cells in culture. Both leptin and an acid-pulse individually significantly increased relative cell numbers (by 22 and 34% respectively). The combination of the two was synergistic (cell numbers 65% above control). The increase in cell numbers in all situations was blocked by the PI3-kinase inhibitor LY294002 (figure 4a). Similar results were seen if proliferation was measured using the BrdU incorporation assay: in both OE33 cells (fig 4b) and OE19 cells (fig 4c), the combination of acid followed by leptin (71% and 52% above basal respectively in the two cell lines) was significantly greater than the effects of acid alone (19% and 11%) and leptin (29% and 26%). All both cases the effect was greater than an additive effect.

**Figure 5:**
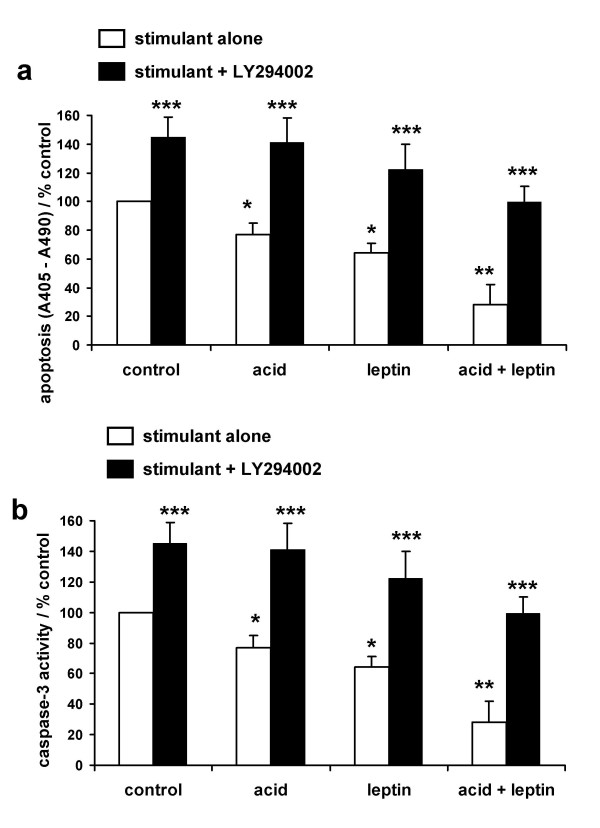
**Akt activation is essential for the anti-apoptotic effects of acid and leptin in oesophageal cancer cells**. Serum-starved OE33 cells were treated with a pH 4.0 acid or control pulse for three minutes and/or leptin (10 nM) for 24 hours and then apoptosis quantified by an ELISA for intracellular nucleosomes (**5a**) or caspase-3 activity in cell lysates (**5b**). Where indicated cells were pretreated with the PI3-kinase inhibitor LY294002 (10 μM) for 60 minutes before any stimulation. Nucleosomes results are expressed as (A_405nm _– A_490nm_) values, which represent relative nuclesome concentrations, compared to untreated control cells. Caspase-3 activity is expresses as percentage of untreated control activity. Results expressed as mean ± SEM, N = 3, * *P *< 0.05 vs untreated control, ** *P *< 0.05 vs acid or leptin alone, *** *P *< 0.05 vs stimulus in the absence of LY294004

Using the assay for intracellular nucleosomes, an acid-pulse alone significantly reduced serum-starvation induced apoptosis by 23% and leptin reduced apoptosis by 36%. Again the combination was synergistic, reducing apoptosis by 72% (fig 5a). These anti-apoptotic actions were also abolished by pre-treatment with LY294002. The PI3-kinase inhibitor itself increased basal apoptosis although it had no effect on basal proliferation. To confirm these results we quantified apoptosis using an alternative method: very similar results were obtained by measuring caspase-3 activity (fig 5b). Acid alone (18% below basal) and leptin alone (22% below basal) reduced apoptosis and the combination was more effective than either alone (76% below basal).

**Figure 6:**
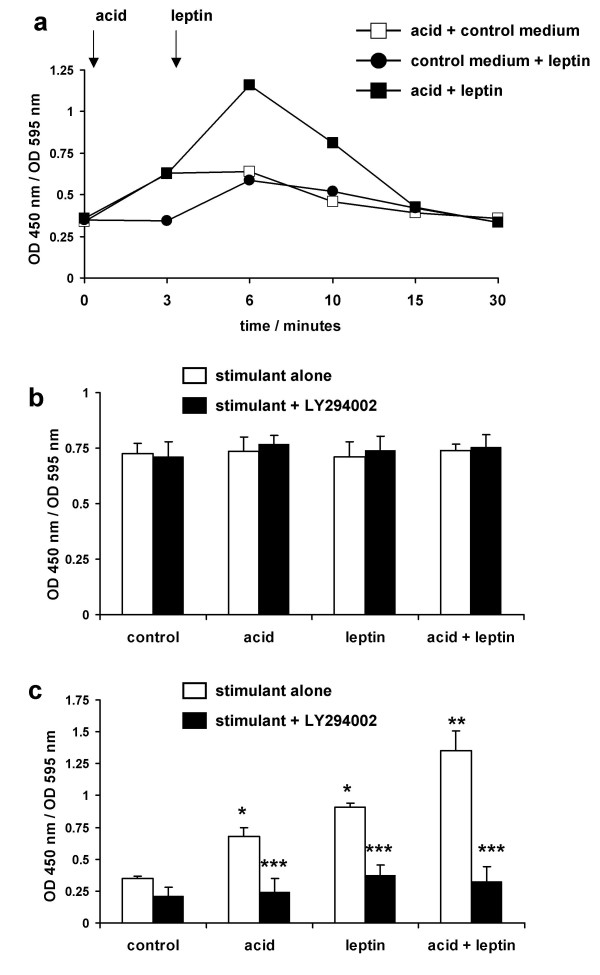
**Effect of acid and leptin on phosphorylation of Akt**. Serum-starved OE33 cells were stimulated with either pH 4.0 acid or control bolus for 3 minutes followed by fresh serum-free medium with or without leptin (10 nM). Cells were formalin-fixed and phospho-Akt and total Akt quantified using cell-based ELISA assays. **6a: time course of Akt phosphorylation**. Serum-starved cells were treated with control medium or acid bolus for 3 minutes and then control medium or leptin. Results represent the mean of quadruplicate wells. **6b: effect on total Akt levels. **5 minutes after the addition of leptin or control medium cells were formalin fixed and total Akt levels quantified. **6c: effect on phospho-Akt levels**. 5 minutes after the addition of leptin or control medium cells were formalin fixed and phsopho-Akt levels quantified. Results expressed as mean ± SEM, N = 3. OD450 nm/OD 595 nm values represent specific reactivity of the peptide of interest in the ELISA corrected for the number of viable cells in the relevant well. * *P *< 0.05 vs untreated control, ** *P *< 0.05 vs acid or leptin alone, *** *P *< 0.05 vs stimulus in the absence of LY294004.

To confirm the activation of Akt, we then examined the effects of acid and leptin on Akt phosphorylation in OE33 cells. An initial time course study showed that acid bolus stimulated an increase in Akt phosphorylation and that levels had returned to basal by 30 minutes. Similarly leptin alone increased Akt phosphorylation, with levels again returning to basal by 30 minutes. Following the acid-bolus with leptin further increased Akt phosphorylation (fig 6a). Detailed quantitation of the effects of acid and leptin showed that there was no change in total Akt levels under any condition (fig 6b) but that acid alone (93% above basal) and leptin alone (159% above basal) significantly activated Akt and that the combination of acid and leptin (287% above basal) was significantly greater than either alone (fig 6c). LY294002 blocked all of these Akt phosphorylating activities (Fig 6c).

**Figure 7:**
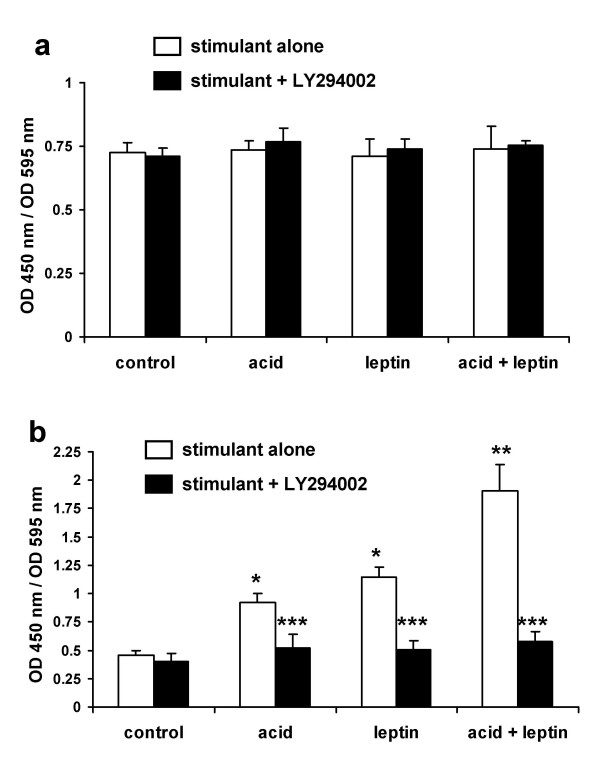
**Effect of acid and leptin on phosphorylation of Bad**. Serum-starved OE33 cells were stimulated with either pH 4.0 acid or control bolus for 3 minutes followed by fresh serum-free medium with or without leptin (10 nM). Cells were formalin fixed 5 minutes after addition of leptin-containing medium for Bad quantitation. Where indicated LY294004 10 μM was added 60 minutes before any stimulation. Phosphorylated and non-phosporylated total Bad were quantified in formalin-fixed cells using ELISA assays. **7a: **effect on total Bad. **7b: **effect on phsopho-Bad. Results expressed as mean ± SEM, N = 3. OD450 nm/OD 595 nm values represent specific reactivity of the peptide of interest in the ELISA corrected for the number of viable cells in the relevant well. * *P *< 0.05 vs untreated control, ** *P *< 0.05 vs acid or leptin alone, *** *P *< 0.05 vs stimulus in the absence of LY294004.

**Figure 8:**
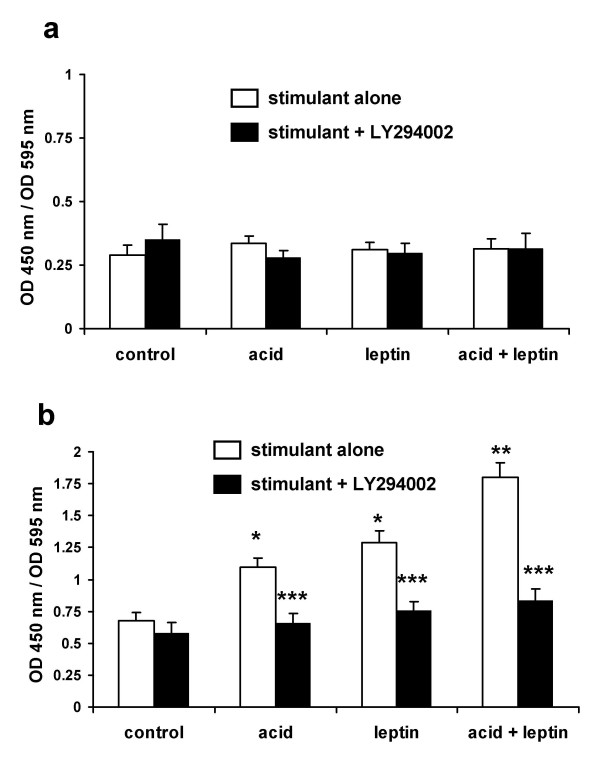
**Effect of acid and leptin on phosphorylation of FOXO1**. Serum-starved OE33 cells were stimulated with either pH 4.0 acid or control bolus for 3 minutes followed by fresh serum-free medium with or without leptin (10 nM). Cells were formalin fixed 5 minutes after addition of leptin-containing medium for FOXO1 quantitation. Where indicated LY294004 10 μM was added 60 minutes before any stimulation. Phosphorylated and non-phosporylated total FOXO1 were quantified in formalin-fixed cells using ELISA assays. **8a: **effect on total FOXO1.**8b: **effect on phsopho-FOXO1. Results expressed as mean ± SEM, N = 3. OD450 nm/OD 595 nm values represent specific reactivity of the peptide of interest in the ELISA corrected for the number of viable cells in the relevant well. * *P *< 0.05 vs untreated control, ** *P *< 0.05 vs acid or leptin alone, *** *P *< 0.05 vs stimulus in the absence of LY294004.

To further characterise the downstream effectors of Akt activation in Barrett's oesophagus we examined the effects of acid and leptin on the phosphorylation of the pro-apoptotic protein Bad and the FKHR transcription factor FOXO1. An acid-bolus, leptin or the combination had no effect on total Bad or FOXO1 levels (figs 7a &8a). Acid and leptin significantly increased phosphorylation of both Bad (fig 7b) and FOXO1 (fig 8b) in a PI3-kinase/Akt-dependant manner. Again the degree of phosphorylation by the combination of acid and leptin was significantly greater than either stimulus alone (figures 7b &8b)

## Discussion

We have described qualitative and quantitative increases in Akt activation in Barrett's oesophagus, high grade dysplasia and adenocarcinoma. Firstly, both HGD and OAC were associated with a very different pattern of Akt activation: with extensive phospho-Akt activation throughout all areas of the epithelium and not just limited to the basal zone, where cell proliferation is typical in normal epithelia [12]. Secondly the density of Akt activation in the crypts increased moving from squamous epithelium to non-dysplastic BO to HGD and adenocarcinoma. These results are consistent with, and extend, the one previous study of Akt activation in Barrett's oesophagus: Harris *et al *demonstrated phospho-Akt staining again limited to the crypts in the basal third of the epithelium in 8 patients with non-dysplastic Barrett's oesophagus with uniform staining for total Akt. However these authors did not investigate the cellular localisation or compare Akt activation with dysplastic or malignant Barrett's [12]. Our current study shows that Akt activation is greater in HGD than non-dysplastic Barrett's oesophagus, supporting a role of Akt in the pathogenesis of Barrett's adenocarcinoma.

Our *in vitro *studies have confirmed that Akt is an important mediator of growth promoting and anti-apoptotic signals in OAC, suggesting that the increased Akt activation demonstrated by the immunohistochemistry is biologically important. One drawback of biopsy-based screening for HGD is the possibility of sampling error [4]. The observation that phospho-Akt staining reached the lumen in all cases, suggests that exploration of the role of detection of phospho-Akt in specimens from brush cytology of the whole oesophagus or upper gastrointestinal liquid aspirates might be a better way of sampling the whole Barrett's segment [37,38]. Similarly, we have studied a relatively small number of patients and it will be important to ensure these findings are replicated in a larger cohort.

The factors driving the increased Akt activation *in vivo *remain to be determined; although it is interesting that *in vitro *both acid exposure and leptin increased Akt activation and the combination was synergistic. These factors may be very important *in vivo*, and of course are consistent with the epidemiological risk factors but our data do not allow us to directly extrapolate to the *in vivo *situation demonstrated in our histopathological study and other stimulants may also be important in driving Akt activation.

It is believed that Akt activation in most cases requires initial phosphorylation of membrane phospholipids by PI3-kinase, this provides a binding recognition site for Akt and this promotes localisation of Akt to the cell membrane, where it is in turn phosphorylated and activated by other kinases, PDK1 appears to be the predominant kinase responsible [11,39]. Subsequently active Akt translocates to both the cytosol and nucleus. Negative regulation involves the phosphatase PTEN [10]. Our data suggest that in HGD and cancer there is an important localisation specific dysregulation of the Akt regulatory pathways but at present whether this specifically reflects increased activation or decreased deactivation cannot be determined. One study suggested that PTEN mutations were not important in oesophageal carcinogenesis [40], but further investigation of this pathway may be fruitful in understanding the factors leading to progression in BO. The specific factors driving Akt activation *in vivo *remain to be determined. For our functional studies we used short term exposure to pH 4.0 acid (to mimic transient gastric reflux and leptin (as a hormone secreted by adipose tissue) as representatives of the two main epidemiological risk factors (reflux and obesity), although other factors could be equally important *in vivo*.

We have shown that oesophageal cancer cells express functional leptin receptors and that leptin stimulated cell growth and inhibited apoptosis. These effects were blocked by LY294002 showing that activation of PI3-kinase links the leptin receptor to Akt activation. We used a LY294002 concentration of 10 μM; previously we have shown that this concentration is effective and specific for inhibiting PI3-kinase-mediated Akt activation in OE33 cells, without any inhibition of extra-cellular signal-related kinase, p38 MAP kinase. c-Jun NH2-terminal kinase, epidermal growth factor kinase, src or protein kinase C [26]. The concentrations of leptin stimulating proliferation and inhibiting apoptosis in OE33 cells are consistent with serum levels in obesity [24]. Proliferative and anti-apoptotic responses of the Barrett's epithelium to the increased serum leptin levels seen in obesity may be an important mechanism in the link between increased body mass index and OAC. Preliminary data have confirmed the presence of leptin receptor isoforms in Barrett's oesophagus and adenocarcinoma: interestingly expression was reported to increase with progression along the dysplasia-carcinoma sequence [25].

The mechanism of acid-induced proliferation is incompletely understood, our data suggest that PI3-kinase-dependant Akt activation in important in mediating the effects of acid exposure. Previous studies have implicated activation of the Na^+^/H^+ ^exchanger, protein kinase C, COX-2 and prostaglandins and the mitogen activated protein kinase cascades in mediating the direct effects of acid [20,21,23,41,42], although it is not clear how these different pathways interact and integrate *in vivo*. To our knowledge this is the first description of acid-induced Akt activation playing a role in Barrett's oesophagus. It is notable that the acid-leptin combination (but not either treatment alone) reduced apoptosis even in LY294002-treated cells with a blocked PI3-kinase/Akt pathway: this suggests that other pathways are activated by the combination and contribute to the anti-apoptotic effects. Further experimental studies exploring the cell signalling interactions of acid and leptin are currently underway.

Further studies will be required to determine how the different signalling pathways activated by acid exposure interact. Epidemiologically oesophageal reflux and obesity are independent risk factors for OAC and the current data suggest that Akt activation might be a point of convergence of these risk factors. Our current data do not allow us to determine the mechanisms of the interaction between leptin and acid. Further detailed studies will be necessary to determine if acid alters the responses to other putative growth factors, alters leptin receptor expression or how signalling converges on Akt activation. In this study we used serum-withdrawal as the apoptotic stimulus, whilst this is a widely used method of examining the putative effects of stimuli and the anti-apoptotic effects of acid in BO have been characterised in serum-starvation experiments [23,43,44], it will be important to establish if leptin and/or acid induced Akt activation has anti-apoptotic actions against other stimuli, such as chemotherapeutic and chemopreventative agents. Leptin has been shown to inhibit serum-starvation and celecoxib-induced apoptosis in HT-29, T84 and Caco-2 colon cancer cells via Akt-dependent pathways [28,45] and inhibit butyrate-induced apoptosis in HT-29 cells, although in the latter study the effect of Akt was not examined [43]. It is believed that reflux of bile acids contributes to the progression of Barrett's adenocarcinoma, although there are less data available on the mechanisms involved compared to acid alone. The bile acid glycochenodeoxycholic acid stimulated proliferation in a non-malignant Barrett's cell line in culture [42]: this effect was shown to be dependent on activation of the extra-cellular signal related kinase and p38 mitogen activated protein kinase pathways. The involvement of Akt was not examined and it will be very interesting to examine the interactions of acid, leptin and bile acids on Akt activation.

We demonstrated that *in vitro *Akt activation lead to phosphorylation of both the pro-apoptotic protein Bad and the FOXO1 transcription factor. These were utilised both as downstream readouts to confirm the functional integrity of the Akt pathway and to provisionally examine whether these pathways might be involved in OAC.

Phosphorylation of cytoplasmic Bad promotes cell survival by inhibiting the mitochondrial apoptotic pathway [29,30]. However our imunocytochemistry data have shown increased nuclear and cytoplasmic localisation of active Akt, suggesting that nuclear targets are important in BO and OAC and Bad is a cytosolic protein. Recently phosphorylation of Forkhead-family transcription factors by Akt has been described. These are initially sited in the nucleus but once phosphorylated, they translocate to the cytosol, where they are transcriptionally inactive [31,32]. This has been proposed to be an important downstream effect mediating the cell proliferative effects of Akt pathway stimulation [46]. We suggest that Forkhead transcription factors are important nuclear targets of leptin and acid mediated Akt activation in Barrett's oesophagus. This is the first description of acid-induced phosphorylation of Bad and FKHR family transcription factors as downstream effects of transient acid exposure. Further immunohistochemistry and *in vitro *studies investigating the activation and functional roles of Bad and Forkhead-family factors in BO and OAC are required to confirm or refute these hypotheses as well as define further downstream Akt targets but both of these would potentially provide new targets for both chemotherapeutic intervention and improved diagnosis.

## Conclusion

In conclusion we have shown increased activation of Akt in Barrett's oesophagus, high grade dysplasia and oesophageal adenocarcinoma. This takes the form of increased Akt activation in the basal epithelium along the progression from squamous epithelium to non-dysplastic Barrett's oesophagus with a further increase with the development of high grade dysplasia and adenocarcinoma. Akt activation is increased in the superficial epithelial zone in HGD and cancers. The obesity hormone leptin and short term acid bolus exposure activate Akt in OAC cells *in vitro *and Akt is essential to the anti-apoptotic and cell proliferative effects. The downstream effects of Akt may be mediated by phosphorylation of Bad and Forkhead transcription factors.

## Abbreviations

BO, Barrett's oesophagus; ELISA, enzyme-linked immunosorbent assay; FKHR, Forkhead; HGD, high grade dysplasia; MTT, 3-[4, 5-dimethylthiazol-2-y-l]-2, 5-diphenyltetrazolium bromide; OAC, oesophageal adenocarcinoma; PCR, polymerase chain reaction; PBS, phosphate buffered saline pH 7.4; PI3-kinase, phosphatidylinositol 3'-kinase; RT, reverse transcriptase.

## Competing interests

The author(s) declare that they have no competing interests.

## Authors' contributions

ILPB initiated the concept and design of the overall study, performed the kinase phosphorylation assays, performed the image analysis, performed the analysis, statistics and interpretation of the data and wrote the original and final versions of the manuscript. OO designed and performed all the experiments using cultured cells. EC participated in the conception and design of the histopathological study and completed recruitment of all subjects. KEA performed analysis and interpretation of the histopathology. GM participated in the immunocytochemistry and PCR and provided support and discussion during design of the studies. MW performed analysis and interpretation of the histopathology and participated in the conception of the study. All authors have approved the final version of the manuscript.

## Pre-publication history

The pre-publication history for this paper can be accessed here:



## References

[B1] Jemal A, Murray T, Ward E, Samuels A, Tiwari RC, Ghafoor A, Feuer EJ, Thun MJ (2006). Cancer statistics, 2005. CA Cancer J Clin.

[B2] Buttar NS, Wang KK (2004). Mechanisms of disease: Carcinogenesis in Barrett's esophagus. Nat Clin Pract Gastroenterol Hepatol.

[B3] Fitzgerald RC (2005). Barrett's oesophagus and oesophageal adenocarcinoma: how does acid interfere with cell proliferation and differentiation?. Gut.

[B4] Wang KK, Wongkeesong M, Buttar NS (2005). American Gastroenterological Association technical review on the role of the gastroenterologist in the management of esophageal carcinoma. Gastroenterology.

[B5] Alikhan M, Rex D, Khan A, Rahmani E, Cummings O, Ulbright TM (1999). Variable pathologic interpretation of columnar lined esophagus by general pathologists in community practice. Gastrointest Endosc.

[B6] Provenzale D, Schmitt C, Wong JB (1999). Barrett's esophagus: a new look at surveillance based on emerging estimates of cancer risk. Am J Gastroenterol.

[B7] Sharma P, Sidorenko EI (2005). Are screening and surveillance for Barrett's oesophagus really worthwhile?. Gut.

[B8] Shaheen NJ, Provenzale D, Sandler RS (2002). Upper endoscopy as a screening and surveillance tool in esophageal adenocarcinoma: a review of the evidence. Am J Gastroenterol.

[B9] McManus DT, Olaru A, Meltzer SJ (2004). Biomarkers of esophageal adenocarcinoma and Barrett's esophagus. Cancer Res.

[B10] Kandel ES, Hay N (1999). The regulation and activities of the multifunctional serine/threonine kinase Akt/PKB. Exp Cell Res.

[B11] Thompson JE, Thompson CB (2004). Putting the rap on Akt. J Clin Oncol.

[B12] Harris JC, Clarke PA, Awan A, Jankowski J, Watson SA (2004). An antiapoptotic role for gastrin and the gastrin/CCK-2 receptor in Barrett's esophagus. Cancer Res.

[B13] Haigh CR, Attwood SE, Thompson DG, Jankowski JA, Kirton CM, Pritchard DM, Varro A, Dimaline R (2003). Gastrin induces proliferation in Barrett's metaplasia through activation of the CCK2 receptor. Gastroenterology.

[B14] Vona-Davis L, Frankenberry K, Cunningham C, Riggs DR, Jackson BJ, Szwerc MF, McFadden DW (2005). MAPK and PI3K inhibition reduces proliferation of Barrett's adenocarcinoma in vitro. J Surg Res.

[B15] Tselepis C, Morris CD, Wakelin D, Hardy R, Perry I, Luong QT, Harper E, Harrison R, Attwood SE, Jankowski JA (2003). Upregulation of the oncogene c-myc in Barrett's adenocarcinoma: induction of c-myc by acidified bile acid in vitro. Gut.

[B16] Lagergren J, Bergstrom R, Nyren O (1999). Association between body mass and adenocarcinoma of the esophagus and gastric cardia. Ann Intern Med.

[B17] Lagergren J, Bergstrom R, Lindgren A, Nyren O (1999). Symptomatic gastroesophageal reflux as a risk factor for esophageal adenocarcinoma. N Engl J Med.

[B18] Caygill CP, Johnston DA, Lopez M, Johnston BJ, Watson A, Reed PI, Hill MJ (2002). Lifestyle factors and Barrett's esophagus. Am J Gastroenterol.

[B19] Engel LS, Chow WH, Vaughan TL, Gammon MD, Risch HA, Stanford JL, Schoenberg JB, Mayne ST, Dubrow R, Rotterdam H, West AB, Blaser M, Blot WJ, Gail MH, Fraumeni JF (2003). Population attributable risks of esophageal and gastric cancers. J Natl Cancer Inst.

[B20] Sarosi GA, Jaiswal K, Herndon E, Lopez-Guzman C, Spechler SJ, Souza RF (2005). Acid increases MAPK-mediated proliferation in Barrett's esophageal adenocarcinoma cells via intracellular acidification through a Cl-/HCO3- exchanger. Am J Physiol Gastrointest Liver Physiol.

[B21] Kaur BS, Triadafilopoulos G (2002). Acid- and bile-induced PGE(2) release and hyperproliferation in Barrett's esophagus are COX-2 and PKC-epsilon dependent. Am J Physiol Gastrointest Liver Physiol.

[B22] Fitzgerald RC, Omary MB, Triadafilopoulos G (1996). Dynamic effects of acid on Barrett's esophagus. An ex vivo proliferation and differentiation model. J Clin Invest.

[B23] Souza RF, Shewmake K, Terada LS, Spechler SJ (2002). Acid exposure activates the mitogen-activated protein kinase pathways in Barrett's esophagus. Gastroenterology.

[B24] Considine RV, Sinha MK, Heiman ML, Kriauciunas A, Stephens TW, Nyce MR, Ohannesian JP, Marco CC, McKee LJ, Bauer TL, Caro JF (1996). Serum immunoreactive-leptin concentrations in normal-weight and obese humans. N Engl J Med.

[B25] Bodger K, Ahmed S, Pazmany L (2005). Over-expression of the leptin receptor in Barrett's metaplasia and cancer. [Abstract]. Gut.

[B26] Ogunwobi O, Mutungi G, Beales IL (2006). Leptin stimulates proliferation and inhibits apoptosis in Barrett's esophageal adenocarcinoma cells by cyclooxygenase-2-dependent, prostaglandin-E2-mediated transactivation of the epidermal growth factor receptor and c-Jun NH2-terminal kinase activation. Endocrinology.

[B27] Somasundar P, Yu AK, Vona-Davis L, McFadden DW (2003). Differential effects of leptin on cancer in vitro. J Surg Res.

[B28] Ogunwobi OO, Beales IL (2007). The anti-apoptotic and growth stimulatory actions of leptin in human colon cancer cells involves activation of JNK mitogen activated protein kinase, JAK2 and PI3 kinase/Akt. Int J Colorectal Dis.

[B29] Spencer JP, Rice-Evans C, Williams RJ (2003). Modulation of pro-survival Akt/protein kinase B and ERK1/2 signaling cascades by quercetin and its in vivo metabolites underlie their action on neuronal viability. J Biol Chem.

[B30] Datta SR, Dudek H, Tao X, Masters S, Fu H, Gotoh Y, Greenberg ME (1997). Akt phosphorylation of BAD couples survival signals to the cell-intrinsic death machinery. Cell.

[B31] Brunet A, Bonni A, Zigmond MJ, Lin MZ, Juo P, Hu LS, Anderson MJ, Arden KC, Blenis J, Greenberg ME (1999). Akt promotes cell survival by phosphorylating and inhibiting a Forkhead transcription factor. Cell.

[B32] Tang ED, Nunez G, Barr FG, Guan KL (1999). Negative regulation of the forkhead transcription factor FKHR by Akt. J Biol Chem.

[B33] Armstrong D, Bennett JR, Blum AL, Dent J, De Dombal FT, Galmiche JP, Lundell L, Margulies M, Richter JE, Spechler SJ, Tytgat GN, Wallin L (1996). The endoscopic assessment of esophagitis: a progress report on observer agreement. Gastroenterology.

[B34] Beales IL (2004). Gastrin and interleukin-1beta stimulate growth factor secretion from cultured rabbit gastric parietal cells. Life Sci.

[B35] Ogunwobi OO, Beales IL (2006). Glycine-extended gastrin stimulates proliferation and inhibits apoptosis in colon cancer cells via cyclo-oxygenase-independent pathways. Regul Pept.

[B36] Beales IL, Ogunwobi O (2006). Glycine-extended gastrin inhibits apoptosis in colon cancer cells via separate activation of Akt and JNK pathways. Mol Cell Endocrinol.

[B37] Geisinger KR (1995). Endoscopic biopsies and cytologic brushings of the esophagus are diagnostically complementary. Am J Clin Pathol.

[B38] Williams GH, Swinn R, Prevost AT, De Clive-Lowe P, Halsall I, Going JJ, Hales CN, Stoeber K, Middleton SJ (2004). Diagnosis of oesophageal cancer by detection of minichromosome maintenance 5 protein in gastric aspirates. Br J Cancer.

[B39] Arico S, Pattingre S, Bauvy C, Gane P, Barbat A, Codogno P, Ogier-Denis E (2002). Celecoxib induces apoptosis by inhibiting 3-phosphoinositide-dependent protein kinase-1 activity in the human colon cancer HT-29 cell line. J Biol Chem.

[B40] Kulke MH, Odze RD, Thakore KS, Thomas G, Wang H, Loda M, Eng C (2001). Allelic loss of 10q23, the PTEN tumour suppressor gene locus, in Barrett's oesophagus-associated adenocarcinoma. Br J Cancer.

[B41] Souza RF, Shewmake K, Pearson S, Sarosi GA, Feagins LA, Ramirez RD, Terada LS, Spechler SJ (2004). Acid increases proliferation via ERK and p38 MAPK-mediated increases in cyclooxygenase-2 in Barrett's adenocarcinoma cells. Am J Physiol Gastrointest Liver Physiol.

[B42] Jaiswal K, Lopez-Guzman C, Souza RF, Spechler SJ, Sarosi GA (2006). Bile Salt Exposure Increases Proliferation Through p38 and ERK-MAPK Pathways in a Non-Neoplastic Barrett's Cell Line. Am J Physiol Gastrointest Liver Physiol.

[B43] Rouet-Benzineb P, Aparicio T, Guilmeau S, Pouzet C, Descatoire V, Buyse M, Bado A (2004). Leptin counteracts sodium butyrate-induced apoptosis in human colon cancer HT-29 cells via NF-kappaB signaling. J Biol Chem.

[B44] Todisco A, Ramamoorthy S, Witham T, Pausawasdi N, Srinivasan S, Dickinson CJ, Askari FK, Krametter D (2001). Molecular mechanisms for the antiapoptotic action of gastrin. Am J Physiol Gastrointest Liver Physiol.

[B45] Hoda MR, Keely SJ, Bertelsen LS, Junger WG, Dharmasena D, Barrett KE (2007). Leptin acts as a mitogenic and antiapoptotic factor for colonic cancer cells. Br J Surg.

[B46] Ramamoorthy S, Stepan V, Todisco A (2004). Intracellular mechanisms mediating the anti-apoptotic action of gastrin. Biochem Biophys Res Commun.

